# Montreal Brain Injury Vision Screening Test for General Practitioners

**DOI:** 10.3389/fnhum.2022.858378

**Published:** 2022-07-14

**Authors:** Reza Abbas Farishta, Reza Farivar

**Affiliations:** ^1^McGill Vision Research Unit, Department of Ophthalmology and Vision Sciences, McGill University, Montréal, QC, Canada; ^2^Research Institute of the McGill University Health Centre, McGill University, Montréal, QC, Canada

**Keywords:** TBI, visual system, binocular vision, screening protocol, concussion, neuro-optometry, visual rehabilitation, visual disturbances

## Abstract

Visual disturbances are amongst the most commonly reported symptoms after a traumatic brain injury (TBI) despite vision testing being uncommon at initial clinical evaluation. TBI patients consistently present a wide range of visual complaints, including photophobia, double vision, blurred vision, and loss of vision which can detrimentally affect reading abilities, postural balance, and mobility. In most cases, especially in rural areas, visual disturbances of TBI would have to be diagnosed and assessed by primary care physicians, who lack the specialized training of optometry. Given that TBI patients have a restricted set of visual concerns, an opportunity exists to develop a screening protocol for specialized evaluation by optometrists—one that a primary care physician could comfortably carry out and do so in a short time. Here, we designed a quick screening protocol that assesses the presence of core visual symptoms present post-TBI. The MOBIVIS (Montreal Brain Injury Vision Screening) protocol takes on average 5 min to perform and is composed of only “high-yield” tests that could be performed in the context of a primary care practice and questions most likely to reveal symptoms needing further vision care management. The composition of our proposed protocol and questionnaire are explained and discussed in light of existing protocols. Its potential impact and ability to shape a better collaboration and an integrative approach in the management of mild TBI (mTBI) patients is also discussed.

## Introduction

Visual disturbances are amongst the most commonly reported symptoms after a traumatic brain injury (TBI) (Armstrong, 2018), but vision testing is uncommon at initial clinical evaluation (typically done by a family physician). TBI patients consistently present a wide range of visual complaints, including photophobia, double vision, blurred vision, loss of vision (Goodrich et al., 2007; Alvarez et al., 2012; Capo-Aponte et al., 2017) as well as other visual processing dysfunctions which can impact fundamental capacities highly reliant on visual processes such as reading, spatial awareness and localization, postural balance and mobility (Kapoor and Ciuffreda, 2002; Magone et al., 2014). Such symptoms are expressed with an estimated frequency ranging widely between 30 and 90% (Ciuffreda et al., 2007; Taub et al., 2012), depending upon the specific criteria used. A proper screening for referral to a trained vision care professional such as a neuro-optometrist who is an optometrist specialized in vision rehabilitation post-TBI, is badly needed to ensure that visual disturbances do not impede recovery, given the central importance of vision and vision comfort to daily function.

Vision care after TBI is rare. This is partly explained by the relative scarcity and uneven access to a qualified eye care professional. In the US, there are ≈16 optometrists for every 100,000 persons in urban cities, 6/100,000 in rural areas and more importantly, 25% of US counties do not have even one optometrist (Feng et al., 2020). As a comparison, primary care physicians and general practitioners (GPs) (Family doctors, general pediatricians) are three times more present across the US, with a distribution of 50/100,000 while only 5% of US counties do not have a primary care physician (Andrilla et al., 2017). This suggests that in most cases, especially in rural areas, visual disturbances of TBI would have to be diagnosed and assessed by primary care physicians, who lack the specialized training of optometry. Given the relative scarcity of optometrist, the physician's judgement of patients' need for optometric evaluation is key to better rehabilitation after TBI. Given that TBI patients have a restricted set of visual concerns, an opportunity exists to develop a screening protocol for specialized evaluation by optometrists—one that a primary care physician could comfortably carry out and do so in a short time.

Here, we identified visual symptoms and signs most frequently reported after mTBI and analyzed known existing vision-related screening and diagnostic protocols as well as self-reported questionnaires (Table 1). Tests and questionnaires proposed in these protocols were assessed for their sensitivity in revealing individuals needing prompt visual rehabilitation and referral to an optometrist. Selecting only “high-yield” test that could be performed in the context of a primary care practice and questions most likely to reveal symptoms needing further vision care management (Ciuffreda and Ludlam, 2011), we have built a screening protocol which would take ≈5 min to complete. Below, we present an overview of vision related TBI screening and diagnostic protocols as well as a brief overview of the main visual functions disturbed post-TBI. The composition of our proposed protocol and questionnaire will then be explained and discussed in light of existing protocols. The potential impact of our protocol and its ability to shape a better collaboration for the care of mTBI patients and an integrative approach in the management of these patients will also be discussed.

**Table 1:** Existing post-TBI vision examination protocol.

**Resource**	**Type**	**Target readership**	**Content**	**Estimated time**
Cilo et al. (2010)	Review	Neuro-optometrist and Neuro-Ophthalmologist practicing in a rehabilitation hospital	Complete ocular exam protocol	1 h
Laukkanen et al. (2017)	Questionnaire	Optometrist performing post TBI evaluation for visual symptoms	28 questions	15 min
Ciuffreda and Ludlam (2011)	Diagnostic protocol	Optometrist for TBI patients	Broad roadmap of testing required during mTBI	1–2 h
Radomski et al. (2014)	Screening protocol	Occupational Therapist involved in the management of service member presenting mTBI with visual sequelae	15-point visual testing protocol	1 h
Goodrich et al. (2013)	Screening protocol	Optometrist for TBI patients	Screening protocol for TBI patients derived from a Delphi study	30 min

## Review of the Most Common Visual Complaints, Systems Affected, and Current Optometric Protocols for TBI Patients

### Most Common Visual Complaints, Systems Affected

**Figure 1:**
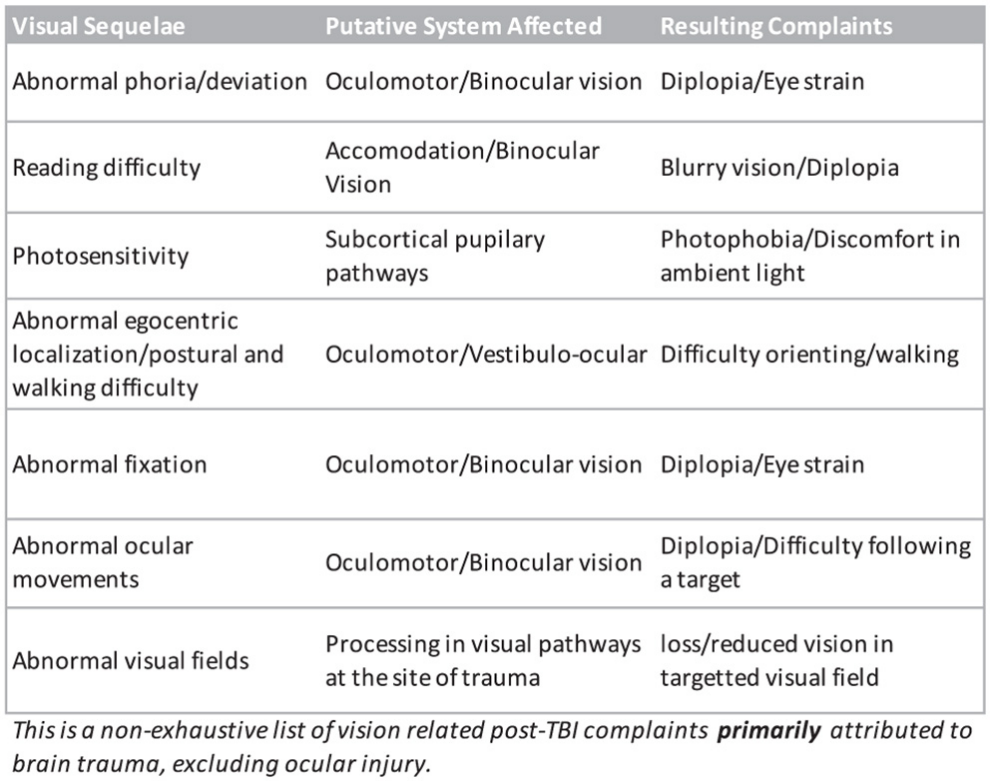
Non-exhaustive list of known visual disturbances reported following a traumatic brain injury.

Mild TBI patients may complain of a plethora of symptoms (Figure 1) depending on the nature of the trauma and the localization and extent of the underlying neural damage but there are core recurring visual symptoms that are reported often by patients (Ripley and Politzer, 2010; Sussman et al., 2016). Some of the most common reported visual complaints are reading difficulty, double vision, eye strain especially when converging, light sensitivity and abnormal spatial localization (Stelmack et al., 2009; Alvarez et al., 2012; Capo-Aponte et al., 2017). Because of their recurring nature, their ability to predict an underlying visual system damage, and the relative ease at which they can be tested, assessing the presence of these symptoms represents a major part of our protocol.

The majority of core visual complaints in concussed patients have a binocular vision or an oculo-motor component. Several studies have reported accommodative dysfunction and convergence insufficiency (CI) as the most frequently occurring visual dysfunction in mTBI. A recent meta-analysis and systematic review on the occurrence of visual complaints after an mTBI reported a prevalence of 43.2% for accommodative dysfunction and 37.2% for convergence insufficiency (Merezhinskaya et al., 2019). In the same study, visual field loss after an mTBI had a prevalence of 6.6% while the search was not able to find any case of visual acuity (VA) loss after a mTBI. Similarly, another retrospective analysis of the frequency of visual impairments and dysfunction among mTBI military personnel also yielded comparable results with 78% of patients reporting subjective visual complaints with accommodative (48.4%) and convergence insufficiency (47.5%) being the two most frequent dysfunctions, while no VA loss was also reported (Brahm et al., 2009). Similar results were also reported in adolescent population where accommodative disorders (51%) and convergence insufficiency (49%) were the most prevalent dysfunctions (Master et al., 2016). Accommodative and convergence insufficiency have also been reported as being one of the most recuring visual dysfunctions in concussed patients in several clinical guidelines (Zelinsky, 2007; Green et al., 2010; Ciuffreda and Ludlam, 2011; Suter and Harvey, 2011; Thiagarajan et al., 2011; Ciuffreda et al., 2014; Ventura et al., 2014; Padula et al., 2017; Fox et al., 2019). Not surprisingly, in a Delphi study seeking to design an optometric examination protocol for the evaluation of vision related complaints in concussed patients, testing for accommodative insufficiency (AI) and CI represented the two core components of the protocol with the highest consensus amongst experts for their importance of testing (Goodrich et al., 2013). In an editorial proposing an objective diagnostic vision test protocol for mTBI patient, Ciuffreda and Ludlam presented a series of tests selected based on considerable clinical and laboratory testing which have demonstrated or predicted abnormal results with few false-positives and thus were referred as “high-yield” (Ciuffreda and Ludlam, 2011). Not surprisingly, testing for convergence and accommodation were the first two items in their streamlined diagnostic protocol. Thus, because of their prevalence, their capacity to be used as high-yield biomarkers of mTBI patients (Ciuffreda et al., 2014) and the ease at which they can be tested, CI and AI represent the core our testing protocol (detailed below).

Other visual symptoms and abnormal ocular and visual findings in concussed patients include visual field defects or loss, cranial nerve disorder, pursuit and saccade disorder, and ocular injuries (Rutner et al., 2006; Cockerham et al., 2009), most of which are either rare occurrences or need a slit lamp and/or specialized training for proper diagnosis and are therefore not fully considered for every concussed patient in our quick screening protocol. Visual field testing could however be useful in some cases. Its testing ideally needs threshold type testing using an automated analyzer which are practically only available in optometry, ophthalmology and neurology clinical settings; a rapid tangent screen perimetry or a confrontation visual field testing which are easy-to-use and cost-effective technique that can be performed by a GP could detect significant visual field losses which are much more likely to happen in moderate to severe brain trauma such as a car accident (Bruce et al., 2006) but remain rare in mTBI (Fox et al., 2019; Merezhinskaya et al., 2019). Moreover, while eye movements could potentially be quickly assessed by a GP, cranial nerve (CN) palsies are rarely encountered after a mild TBI with an incidence of 0.3% (Coello et al., 2010). Amongst these rare occurrences, CNI, VII and VIII are most often involved and aren't primarily involved in ocular movements. CN palsies will most likely arise after a severe trauma in which case the patient will most likely have been assessed in an emergency clinical setting. However, should the patient report diplopia in certain gaze position, the GP could promptly identify the symptomatic direction of gaze and refer the patient accordingly to a neurologist.

### Current Optometric Protocol and Resources

Several resources including questionnaires and protocols have been published for optometrists and occupational therapists (OT) for the screening of TBI patients presenting visual sequelae. These resources are presented in Table 1. Most resources could be categorized in two broad groups: the first group consists of review papers mainly designed for optometrists or specialized health care professionals involved in the visual rehabilitation of mTBI patients; these papers were mostly detailed reviews of the literature highlighting the possible ways in which components of the visual system could be influenced by a TBI. Some also include broad visual testing procedures and vision therapy rehabilitation strategies (Zelinsky, 2007; Cilo et al., 2010; Sussman et al., 2016; Fox et al., 2019). While informative for their intended readership, these reviews did not address the main goal of our proposed protocol—to design a fast and efficient tool for GPs to screen potential concussed patients needing further visual testing. The second group of papers was perhaps of greater interest since it consisted of screening and diagnostic protocols designed for the clinical management of concussed patients presenting visual sequelae. They differed from the first group in that they did not only highlight possible visual functions affected in mTBI patient, but proposed a targeted screening and diagnostic routine, either based on clinical expertise and/or known prevalence of mTBI visual symptoms and dysfunctions (Ciuffreda and Ludlam, 2011; Goodrich et al., 2013; Radomski et al., 2014). While clinical reasoning and evidence-based studies were cited to justify the selection of each item, the efficacy of these diagnostic protocol has not been validated. Out of all protocols listed in Table 1, only two were designed using a standardized method, namely using a Delphi approach and a consensus based nominal group technique (Goodrich et al., 2013; Radomski et al., 2014). The main difference between these protocols and ours is that they were designed for optometrists (Goodrich et al., 2013) or for OTs (Radomski et al., 2014) either working with mTBI patients or willing to specialize in their clinical management which made them unsuitable of non-optometrists practitioners for three main reasons: first, visual testing routine included tests regardless of their feasibility by a non-optometrist practitioner and hence, a significant portion of their protocol became irrelevant for GPs; second, they included tests which needed material whose cost and specificity of use would make it a very unlikely tool to be used by a non-optometrist, regardless of their ease of use. Third, because these protocols were targeted for optometrist involved in the rehabilitation of mTBI patients, they consisted of a battery of tests also used as benchmark to evaluate follow-up visits and vision-therapy intervention effect. In doing so, these protocols were mostly long and meticulous which made them non suitable for quick screening purposes.

The recurring nature of vision related TBI sequalae means that most resources presented a high degree of consistency and had similar procedures with only minor differences in the method of testing. The only two testing protocol designed using a standardized method mentioned above also presented high degree of similarity (Goodrich et al., 2013; Radomski et al., 2014). Both screening protocol targeted limited items (7 and 9) out of which six were shared: (1) binocular alignment; (2) ocular motility or pursuit; (3) saccades; (4) accommodation; (5) near point of convergence; (6) self-reported questionnaire. The Delphi study, which was designed for optometrists, conducted by a panel of optometrists, and based on a methodical consultation of optometrists involved in TBI rehabilitation had only one added item other than the six mentioned above: a repeated Near point of convergence test, which is done to confirm results of an initial NPC and to test the system's capacity to engage a repeated effort over time. The protocol using a nominal group technique was primarily designed for occupational therapists involved in the visual rehabilitation of TBI patients which included mild and moderate TBI, unlike the Delphi study which only targeted mTBI patients. Other than the six shared items, this protocol also included far and near visual acuity and visual field testing. This difference is likely due to the fact that VA and visual field losses are much more likely to happen in moderate to severe TBI (Fox et al., 2019; Merezhinskaya et al., 2019) and also because OT's role in the clinical management of TBI patient during their rehabilitation is done to improve function and to maintain autonomy and meaningful activities for which visual field assessment remains crucial. Both protocols included a questionnaire for TBI patients: Radomski and colleagues proposed OTs to use the graded questionnaire on the quality of life developed by the college of optometrists in vision development (COVD) (Daugherty et al., 2007) while in their Delphi study, Goodrich et al. also used their survey to select 17 questions which are important to ask to a concussed patient experiencing visual discomfort. Out of them, five questions targeted the medical history of the patient and circumstances surrounding the trauma (localization, time, etc.); the remaining 12 questions targeted current symptoms and targeted four core complaints or disturbed activity: (1) mobility, (2) reading ability, (3) blurry or double vision, and (4) photophobia and headaches. A more recent paper also proposed a graded questionnaire specifically designed to screen and evaluate the rehabilitation of TBI patients (Laukkanen et al., 2017). Comprised of 28 graded questions roughly targeting core complaints of TBI patients, the Brain Injury Vision Symptoms Survey (BIVSS) is the only validated vision specific questionnaire designed for mTBI patients. This questionnaire has now become a standard amongst optometrists for the clinical management and rehabilitation of mTBI patients presenting a sensitivity of 82.2% for correctly predicting TBI from control subjects. Because of its known validity, we have mostly used graded questions from the BIVSS in our own protocol.

## Our Proposed Protocol

**Figure 2:**
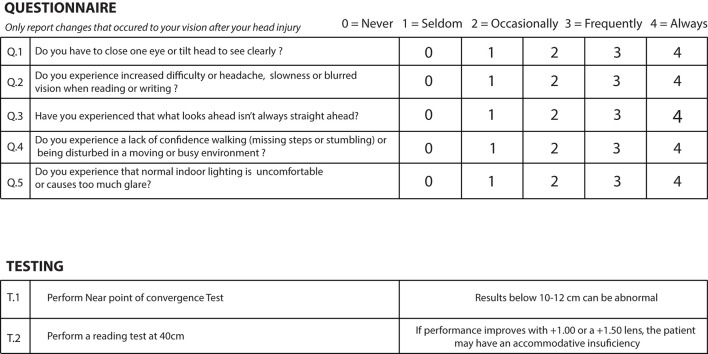
Proposed visual screening protocol.

Like current protocols designed for optometrists, we also sought to design a screening tool composed of quick screening tests to be carried out by the GP and a questionnaire to be completed by the patient (Figure 2). Questions in the questionnaires were selected taking into consideration core visual complaints mentioned above and their likelihood to identify patients requiring further optometric attention. The same criteria were used for the selection of the tests with the only difference that we also made sure testing could be done using low-tech and easy to access material and could be carried out by non-specialized health care professionals.

### Questionnaire

Most existing protocols and clinical review on vision-related TBI symptoms have emphasized the importance of self-reported complaints as patients will often recognize some function loss, especially when reading (Reddy et al., 2020; Pei and O'Brien, 2021), which may often be the reason for a consultation in the first place. These self-reported symptoms are important as they can direct physicians in their referral and highlight the extent of the visual system involvement post-TBI.

Among the existing TBI and vision-related questionnaires available, two are mostly used in clinical optometry: the Brain Injury Vision Symptom Survey (BIVSS) (Laukkanen et al., 2017) which identified questions most likely to discriminate TBI patients from uninjured individuals, and the College of Optometrists in Vision Development (COVD) Quality of Life Outcomes Assessment (Daugherty et al., 2007), which is used amongst optometrist to assess baseline quality of life with regards to visual symptoms. Another questionnaire has also been designed as a clinical guideline for eyecare professionals to be used during the visual examination of mTBI patients (Goodrich et al., 2013) with roughly similar questions than those present in the BIVSS and the COVD questionnaires.

These questionnaires are relatively long with 28, 19, and 17 questions, respectively. They are detailed and require a degree of understanding and participation from the participant which makes them ideal for *follow-up* during post-TBI visual rehabilitation, but impractical to be used for a quick screening protocol performed by a non-specialist healthcare professional. For these reasons, we narrowed down our questions to five, each one directly targeting most frequently occurring core post-mTBI visual complaints to highlight their presence based on meta-analysis driven prevalence or clinical expertise reviews, while keeping the graded system to ensure responses highlighted recurring visual disturbances. We kept the gradation used by the BIVSS which allows the participant to report the intensity of a given complain from 0 to 4 (0 = never; 1 = seldom; 2 = occasionally; 3 = frequently; 4 = always), which was also the same gradation used by the COVD questionnaire. For the 28 items questionnaire, the BIVSS reports that a score of 45 and suggests significant visual disturbance related to TBI symptoms. Since our questionnaire is mostly based on the BIVSS while representing only a sixth of its length, a score of eight and above in our questionnaire could potentially be suggestive of a significant visual discomfort for TBI related symptoms. All questions must be asked specifically by the GP regarding changes perceived after the trauma to avoid registering symptoms that were present before the trauma.

**Figure 3:**
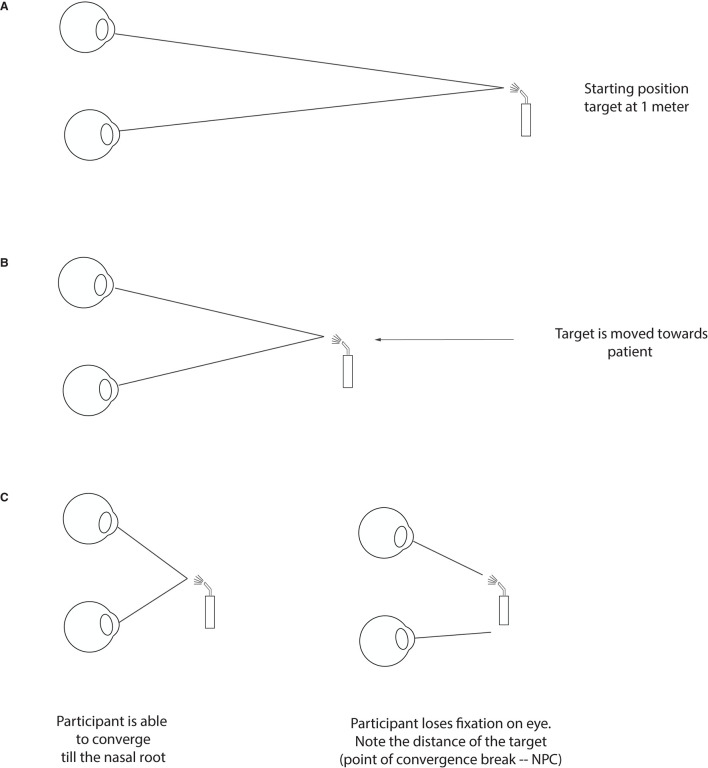
Testing for convergence insufficiency. At the starting position, a target is placed roughly at 60 cm from the patient (about armlength), slightly below the midline **(A)**. The target is then moved slowly and steadily forward toward the bridge of the patient's nose and clear instructions are given to report double vision **(B)**. In the process, the patient will either maintain fixation until their nose or lose fixation (one eye will deviate) at the point of convergence break. The distance at which this break happens (or when the patient reports seeing double) is the NPC and is noted in cm **(C)**.

*Q*1. Binocular vision involvement is very frequent post-TBI and many patients may suffer from convergence insufficiency (CI) (Ciuffreda et al., 2007; Alvarez et al., 2012). These patients may experience eye strain which can be greater upon reading as the need for convergence in near vision work is greater than when looking straight ahead at distance (Figure 3) (Trbovich et al., 2019). When ocular deviation post-TBI is too great for the patient to compensate by straining, the visual system may either suppress one image or the patient may see double. One common strategy when faced with recurring diplopia is the closure of one eye. Since the amount of deviation post-TBI can vary depending on the eye gaze position, some patients may also develop strategies whereby a head tilt may enable them to see better. Our first question was therefore designed to highlight the possible presence of a binocular vision involvement:

*Do you have to close one eye or tilt your head to see clearly?*

**Figure 4:**
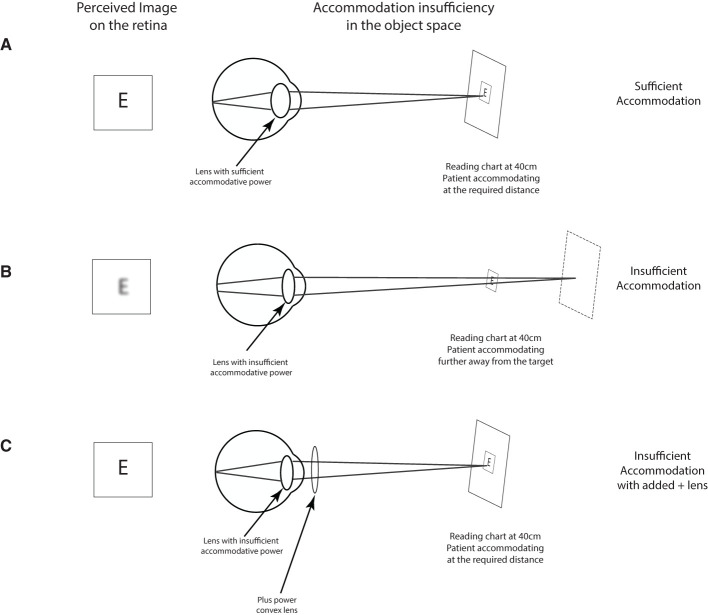
Testing for accommodative insufficiency. Typically, a non-presbyopic patient (typically below 40 years of age) should have no problem accommodating on a reading target placed at 40 cm **(A)**. In the presence of an accommodative insufficiency, the patient is unable to focus on the target and sees the reading blurry **(B)**. In such a case, the addition of a positive lens (+1.00 to +2.00) should bring the target within the patient's range of accommodation which should bring the target in focus **(C)**.

*Q*2. Another core visual complaint post-TBI has to do with accommodative insufficiency (Ciuffreda et al., 2007; Chen et al., 2020), i.e., the inability of the visual system to accommodate on a visual target (Figure 4). While accommodation is engaged every time we focus on a target, its function can mostly be tested when reading. This is because some level of blur may not impede the recognition of targets such as faces and objects as many cues can be used to recognize them while even little to moderate amount of blur can slow down reading speed for smaller prints (Chung et al., 2007) and would likely impact accurate writing. We have sought to test the presence of accommodative problems by asking the following question:

*Do you experience increased difficulty or headache, slowness or blurred vision when reading or writing?*

*Q*3. TBI patients can also report disruption in their spatial localization especially in relation to their own body (Bansal et al., 2014). This is often referred to as Visual Perceptual Midline Shift Syndrome or Abnormal Egocentric Localization (Padula et al., 2017; Labreche et al., 2020) which may highlight the involvement of oculomotor and vestibulo-ocular systems. Patients suffering from abnormal visual spatial processing may perceive objects that are in line with their vertical center of their body to be shifted on one side. In their validated BIVSS, Laukkanen et al. had found that questions related to perceived spatial localization are amongst the most sensitive in differentiating concussed patients from controls. We therefore included the same question as found in the BIVSS:

*Have you experienced that what looks ahead isn't always straight ahead?*

*Q4*. One of the consequences of the abnormal spatial perception post-TBI mentioned above, is that this perceptual shift may impact a patient's ability when walking and avoiding targets and may also contribute to or be part of the known vestibular problems often encountered post-TBI (Wallace and Lifshitz, 2016; Szczupak et al., 2020). Another symptom with vestibular involvement is an increased visual motion sensitivity (VMS), the fact that concussed patients can be more sensitive in crowded environments due to their increased sensitivity to visual motion (Ciuffreda et al., 2016). Because some of these symptoms can be treated or alleviated with optometric rehabilitation typically with the use of yoked prisms or bi-nasal occlusion (Bansal et al., 2014; Padula et al., 2015), a positive answer to questions 3 and 4 are highly suggestive of a need for referral. We sought to address the mobility of concussed patients with the same question used in the BIVSS and shown to be more specifically targeting post-TBI symptoms.

*Have you, since your injury, noticed lacking confidence walking (missing steps or stumbling) or being disturbed in a moving or busy environments?*

*Q5*. While questions 1–4 had mostly a visuo-motor component, question 5 is primarily targeting the presence of photophobia, which is amongst the most prevalent symptoms post-TBI (Wu and Hallett, 2017) and is a symptom addressed in the BIVSS. Most studies estimate the prevalence of photophobia to be between 30 and 40% in the early stages. Even after a year, 20% of all patients will continue to report disturbance to ambient light (Merezhinskaya et al., 2021). Since these symptoms can be managed by the prescription of tinted glasses (Jackowski et al., 1996; Clark et al., 2017), a positive response to this question should also warrant a referral to a neuro-optometrist. Photophobia and discomfort related to lighting was addressed by asking the following question:

*Do you feel that normal indoor lighting is uncomfortable or causes too much glare?*

### Tests

Along with a subjective questionnaire, we sought to add objective tests that could target core visual dysfunction observed in mTBI patients based on their occurrence rate. As mentioned above convergence (Figure 3) and accommodative (Figure 4) insufficiency have consistently been the most reported visual dysfunction in mTBI patients. Conveniently, these dysfunctions are amongst the easiest to diagnose and detect and require very minimal material and were therefore an ideal inclusion for our protocol.

#### Testing for Convergence Using Near Point of Convergence (NPC)

As mentioned for the Question 1, one of the common visual disturbances of TBI patients is the difficulty with near work typically when reading and doing computer work, especially when patients see double. These symptoms often point to a convergence insufficiency (Figure 3), where the patient is no longer capable of converging their two eyes at the desired target. This lack of adequate convergence generates different and non-fusible images (diplopia) in the visual representation of their two eyes. Fortunately, convergence insufficiency is one of the easiest problems to detect—it is detected by measuring the nearest point of convergence (NPC) which consists in advancing an accommodative target, typically using a penlight, from about 60 cm in front of the patient's primary position eye gaze toward their nose (Figure 3A). As the target gets closer (Figure 3B), the patient's eyes will converge to keep up with the moving target. Most adults and teens can perform this task without any significant problem, and they may be able to either converge till their nose or lose their fixation close to it (Figure 3C). Any value below 10 cm is considered normal range (Hayes et al., 1998; Scheiman et al., 2003).

Post-TBI, a clinician can expect higher values at the NPC such that the nearest convergence point is farther away from the root of nose (Ciuffreda et al., 2007; Master et al., 2016). This means that as soon as the clinician advances the target toward the patient, one of the eyes will stop converging with the approaching target which can be observed by the GP. This break in convergence can be subjectively perceived by patients, as they will report double vision, or they may continue to see one image despite the other eye losing fixation due to suppressive mechanism as the brain will actively suppress the image of the deviating eye to avoid seeing double. Even if suppressive mechanism are present, the GP will still be able to notice the break of fixation objectively. During this test, it is important for the patient to mention if they see double as it helps the clinician locating the point of convergence break; and to report any eye strain as this can also suggest that the system is not able to fully sustain convergence on a near target. Values >10 cm, accompanied with reading difficulties post-TBI, warrants a referral to an optometrist/neuro-optometrist for complete work-up and management.

#### Test 2: Testing for Accommodative Insufficiency

Near vision problem can also arise due to an accommodative problem which can happen in conjunction with convergence problems—accommodation and convergence are tightly linked (Alvarez et al., 2012; Capo-Aponte et al., 2012). An accommodative insufficiency is typically understood as the inability by the visual system to focus on the desired target typically due to lower-than-expected accommodative reserves (Figures 4A,B). This “de-focus” will manifest itself for the patient in a blurred perception. An accommodative insufficiency is tested by assessing the patient's capacity to focus and read using a near vision reading chart at 40 cm. Post-TBI, a clinician can expect reading difficulties during this test (Chen et al., 2020), and the difficulty can be alleviated by use of a positive lens—typically varying from +1 to +2.00 that can be added while the patient is reading (Figure 4C). To dissociate with possible presbyopia, physicians should verify that the blurred perception during reading began *after* the head injury.

## Discussion

We have defined a minimal, easy-to-use, quick protocol to be carried out by a GP over a few minutes that can flag a patient for referral to an optometrist for professional evaluation and treatment. The protocol includes a small number of questions that can inform the GP of the most common visual disturbances after TBI, and two tests that can highlight challenges to convergence and accommodation—both concerns of great importance in this population.

Many of the other common symptoms of TBI—such as headaches and dizziness—may have an ocular or oculo-motor source and could be potentially treated by an optometrist (Kontos et al., 2017; Mucci et al., 2019). This could therefore represent a low-cost and easy path to treatment and recovery of quality of life for many patients. Visual discomfort after TBI seriously limits a person's quality of life, as the bulk of our modern life involves using digital screens and reading fine text, which many TBI patients find difficult to engage with.

Convergence issues are among the most debilitating, as they can contribute to dizziness and even nausea, in addition to diplopia and spatial confusion (Trieu and Lavrich, 2018). Such issues can be hard to detect without testing, as GPs may treat the symptoms of dizziness or nausea as being caused by vestibular dysfunction, without ever testing whether convergence is a potential concern. For this reason, our rapid protocol includes a quick screening test for convergence insufficiency, with a positive outcome demanding referral to an optometrist.

By some estimates, we spend about 8 h a day on digital screens, much of it involving high-acuity work (reading, texting, browsing, etc.) (Bahkir and Grandee, 2020; Alabdulkader, 2021). Accommodative insufficiency thus could prevent a TBI patient from participating in their routine activities. Fortunately, it is both easy to test and easy to correct with positive lenses. Thus, testing for accommodative insufficiency must be an essential aspect of screening for referral to an optometrist.

In this protocol, we have voluntarily used optometrist and neuro-optometrist interchangeably when referring patients whose results were outside the norm. We have done so keeping in mind the various setups and conditions in which GPs may practice, which could impact their referral strategies. As an example, 25% of US counties do not have an optometrist (Feng et al., 2020) and patients residing in these counties may therefore have added difficulty finding a general eye doctor—a referral to a neuro-optometrist only makes the whole process even more difficult. On the other hand, a patient living in an area with good optometric presence will likely find it easier to be referred directly to a neuro-optometrist to get their vision tested and start their rehabilitation process with minimum delays.

While very few studies have investigated the exact timeline of visual disturbances post-mTBI, most reports suggest that they can be classified into acute (symptoms appear after the trauma and within 3 months) and chronic (persistent after 3 months and even after a year) (Masel and DeWitt, 2010; DeKosky et al., 2013). Our screening protocol will mostly likely be useful for patients who have recently suffered from an mTBI and consult a GP for the plethora of symptoms which accompany mTBI including visual ones since we can assume that patients presenting persisting visual symptoms after a year will most likely consult an optometrist without necessarily consulting their GP. Because of the minimum level of cooperation involved in our protocol (filling the questionnaire and performing the tests), our protocol is mostly targeting adults and late adolescent who are more likely to accurately verbalize visualize changes occurring after their trauma.

While we have singled out GPs as the main users of this protocol, the quick and easy nature of its design makes it a very valuable tool to be used by any frontline health care professional in a wide variety of settings. This can include nurses and physicians' assistants and other health care providers likely involved in post-TBI rehabilitation such as physiotherapists and occupational therapists.

Our quick protocol is, by design, limited to visual disturbances that arise following brain injury specifically. In some cases, head trauma can also happen in conjunction with ocular trauma, especially when the injury is close to the ocular globe and orbital region. In such cases, it is very important that a patient be referred to an optometrist for a dilated fundus exam regardless of their answers and results from our protocol as injury to the eye can cause severe complications such as angle recession, dislocated lens, secondary glaucoma or *comotio retinae* (Pelletier et al., 1998; Mufti et al., 2020). Our quick protocol also cannot exclude presbyopia or uncorrected hypermetropia when patients express reading difficulty, except by asking patients for changes to their reading capacity *after* their head injury. This can however be easily tested at the optometrist upon referral.

We specifically designed our protocol targeting mTBI for two reasons. First, because of their higher occurrence rate (Cassidy et al., 2004), mTBI patients are most likely to form the bulk of patients seeking medical attention from a GP in an outpatient setting. Moreover, because of their milder symptoms, they are also most likely to have underdiagnosed visual dysfunctions and hence could greatly benefit from a targeted approach to their visual health. For this reason, our protocol does not formally include other important and easy to perform tests that could be done by a GP that may be relevant to TBI patient suffering from a serious trauma. This includes visual field testing and the evaluation of eye movements. Studies have shown that visual field defects and cranial nerve palsies are very rare in mTBI patients and are more frequent in moderate and severe TBI (Merezhinskaya et al., 2019). We however suggest GPs to investigate this possibility especially in the presence of a trauma caused by a car accident or when the patient reports double vision in specific gaze directions.

Our protocol's ability to become a standard in the field rests on its usability and its sensitivity in referring TBI patients needing prompt vision care. To our knowledge, none of the protocol consulted in Table 1 were validated in a study, which highlights the need of greater investigation in the field. While we acknowledge that this is a limitation of our current protocol, we are confident in its clinical relevance, mostly because its tests and questions were selected based on high level of consensus amongst experts on the importance of testing CI and AI (Goodrich et al., 2013; Radomski et al., 2014), on their possible use as biomarkers (Ciuffreda et al., 2014), and the fact they remain very prevalent amongst TBI patients (Merezhinskaya et al., 2019). Moreover, while available protocols in Table 1 have not been validated, the BIVSS questionnaire has been validated with excellent sensitivity. Since our questionnaire was formulated using the BIVSS and known prevalence of TBI dysfunction occurrence which also featured in the BIVSS we believe that our protocol should be able to attain comparable sensitivity. Our hope is that by sharing this protocol, its validity and usability could be studied in a multitude of setting highlighting the specificity of health care systems in which GPs normally practice.

With the growing awareness of TBI impact on brain health, patients are likely to consult a health care physician with greater frequency. Our growing use of electronic devices, especially the overwhelming presence of screens and near work has made the visual disturbances of TBI even more disruptive to quality of life as they often lead to dropped productivity and increased frustration for the patient (Broshek et al., 2015). Therefore, any strategy geared toward a better and quicker diagnosis leading to a more efficient referral and management of those patients can significantly improve patient care and the public health management of TBI. In this review, we argued that a critical way toward achieving this goal is by increasing GP-Optometrist collaboration by providing efficient tools for GP's to better recognize post TBI visual disturbances and refer them to an optometrist with knowledge of visual rehabilitation processes. We have therefore designed a protocol which on average takes 5 min consisting of a quick questionnaire and two tests testing the binocular function, convergence, and accommodation.

## Author Contributions

Conceptualization and writing—review and editing: RAF and RF. Methodology, review of literature, data curation, and writing—original draft preparation: RAF. Funding acquisition: RF. Both authors have read and agreed to the published version of the manuscript.

## Funding

This work was supported by a Canada Research Chair research stipend to RF.

## Conflict of Interest

The authors declare that the research was conducted in the absence of any commercial or financial relationships that could be construed as a potential conflict of interest.

## Publisher's Note

All claims expressed in this article are solely those of the authors and do not necessarily represent those of their affiliated organizations, or those of the publisher, the editors and the reviewers. Any product that may be evaluated in this article, or claim that may be made by its manufacturer, is not guaranteed or endorsed by the publisher.
